# Supplementary material for: Human skeletal muscle plasmalemma alters its structure to change its Ca2+-handling following heavy-load resistance exercise
Source: Nat Commun. 2017 Feb 13;8:14266. doi: 10.1038/ncomms14266 (PMC5316829; doi:10.1038/ncomms14266)
Supplement: Supplementary Information — Supplementary Figures, Supplementary Tables, Supplementary Methods and Supplementary References [file ncomms14266-s1.pdf]

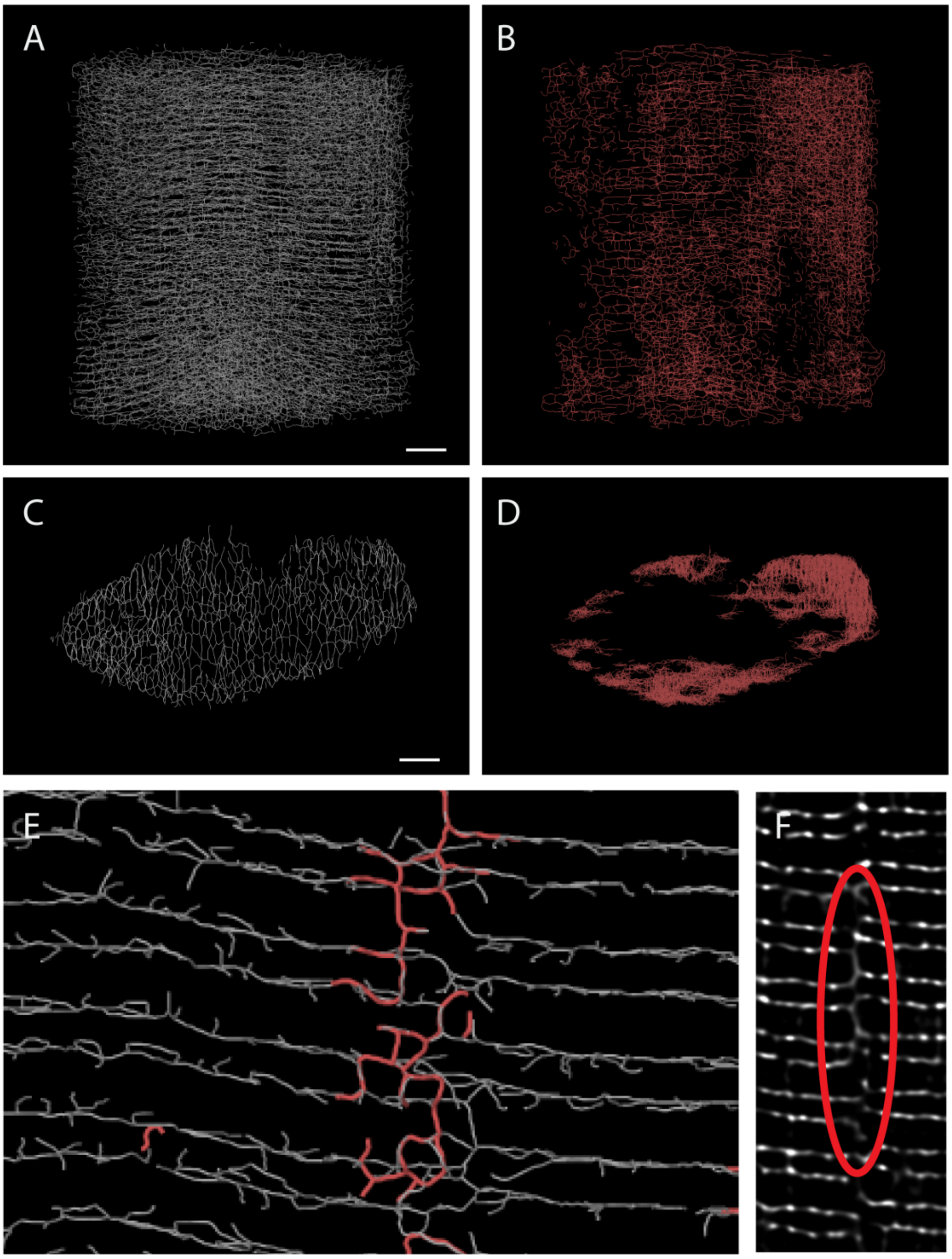

Supplementary Figure 1: 3D skeletonised reconstruction of the human skeletal muscle t-system. (A) An example of a skeletonised t-system reconstructed from an ~ 60 µm deep confocal volume illustrates the full complexity of the t-tubular network. (B) Automated segmentation of all tubules which are either extending over 2 µm parallel to the longitudinal axis of the fibre or directly connected to one revealed the longitudinal networks of the t-system. In comparison to the full transverse network of the t-system (C) visualised in a 2 µm -deep volume in orthogonal view to panel A, the longitudinal networks appear to be primarily localised near the peripheral regions of the fibre's cross-section. Close inspection of the longitudinal tubule network (E) revealed that many longitudinal tubules were located in between myofibrils which were misaligned. (F) in confocal 2D data, these regions showed misregistrations between transverse elements of the t-system (circled). Scale bars: 8 µm.

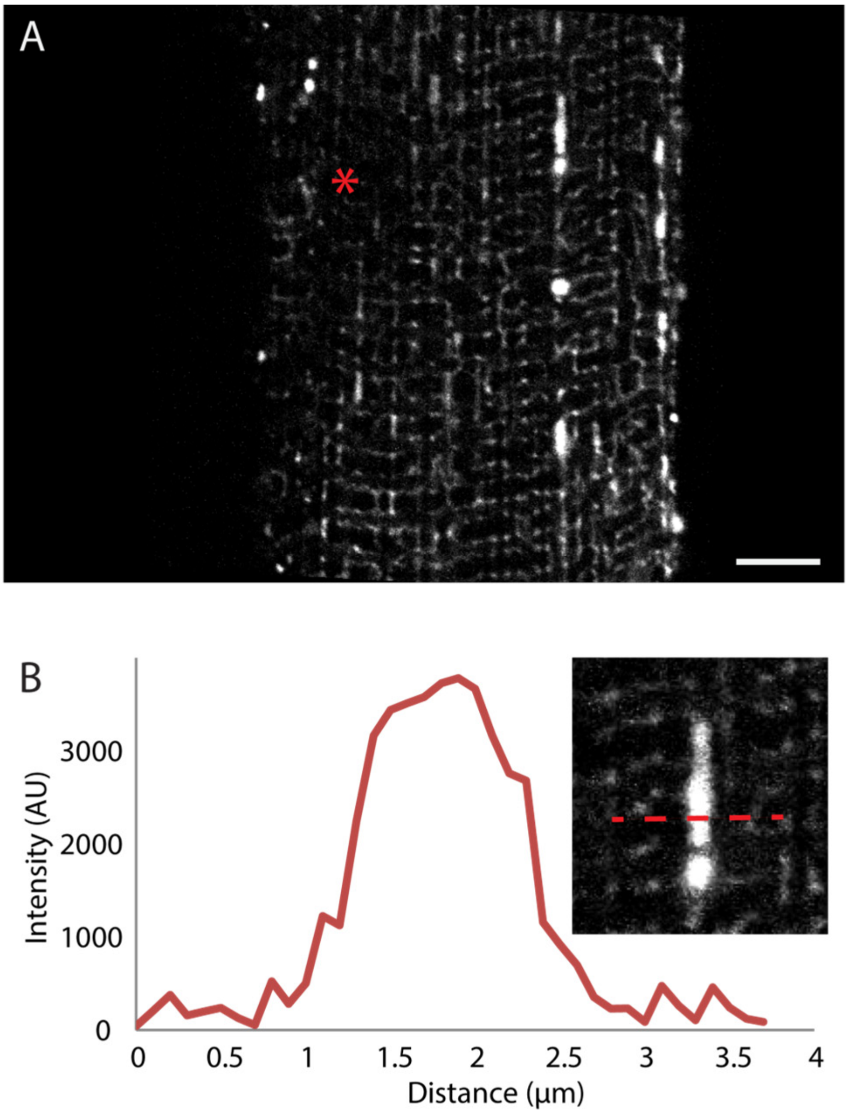

Supplementary Figure 2: Morphological characterisation of the post-exercise t-system. (A) A confocal optical section of a fibre biopsy obtained 24 hours following exercise illustrates the non-uniformity in dye distribution leading to apparently dark regions (asterisk) which are caused by the redistribution of the dye into the vacuoles (bright structures). (B) The approximate diameter of dye-filled vacuoles can be estimated by plotting a line intensity profile bisecting these structures. With a full-width at half-maximum of this intensity profile of 1 µm, these vacuoles appear to be >10-fold wider than the t-tubules measured previously in mammalian skeletal muscle. Scale bar: 5 µm.

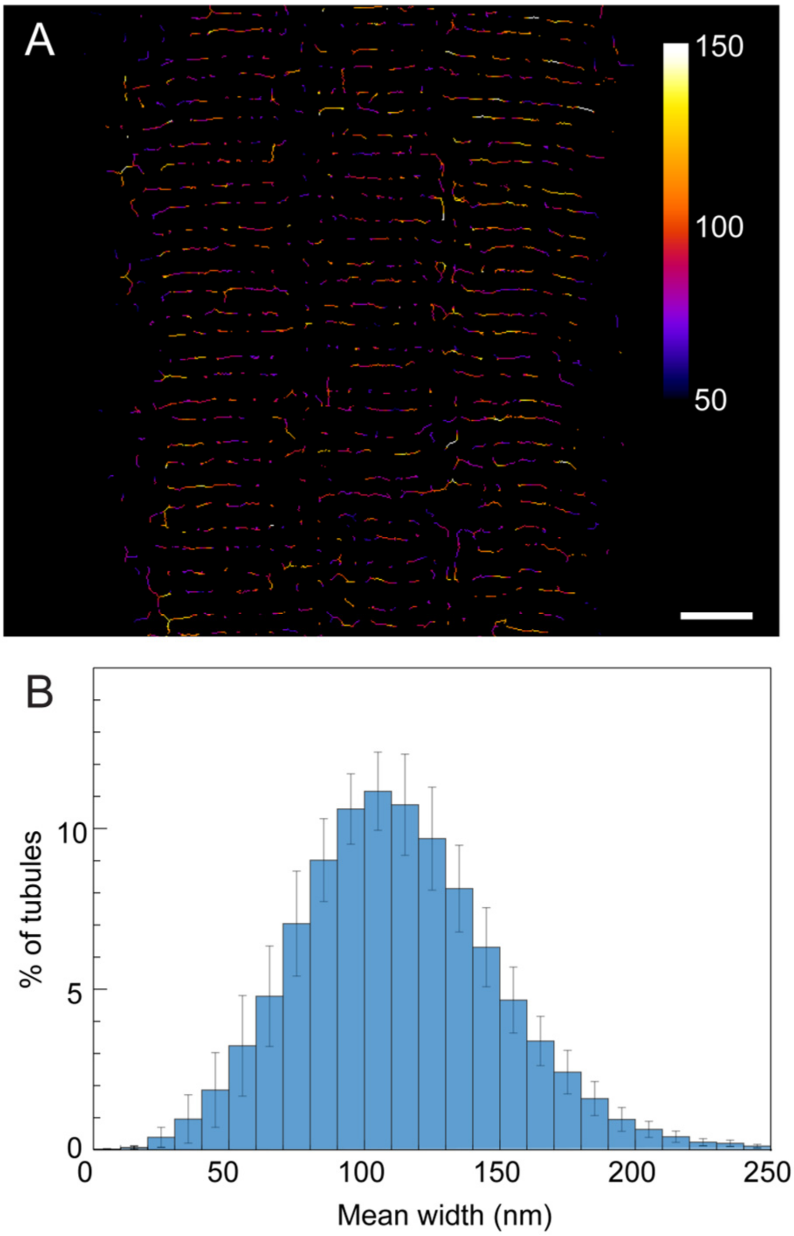

Supplementary Fig 3. Fluorometric calibration of mean local diameter of t-tubules. (A) a 1.5 µm deep projection of the skeleton of a the t-system constructed from deconvolved 3D confocal z-stack is shown colour-coded for the estimated mean local diameter of the tubules. This calculation was made based on the assumptions that skeletal muscle tubules are similar in shape to a flattened cylinder and that the Fluo5N dye and $[Ca^{2+}]_{t\text{-}sys}$ is uniform throughout this compartment. See Jayasinghe & Launikonis[1] for a detailed account of the calibration process and limitations. Colour calibration bar mean tubule width in nanometres. Scale bar: 5 µm. (B) A histogram of the mean tubule widths calculated from human skeletal muscle fibre biopsies prior to exercise. Error bars report SEM (n = 3 fibres).

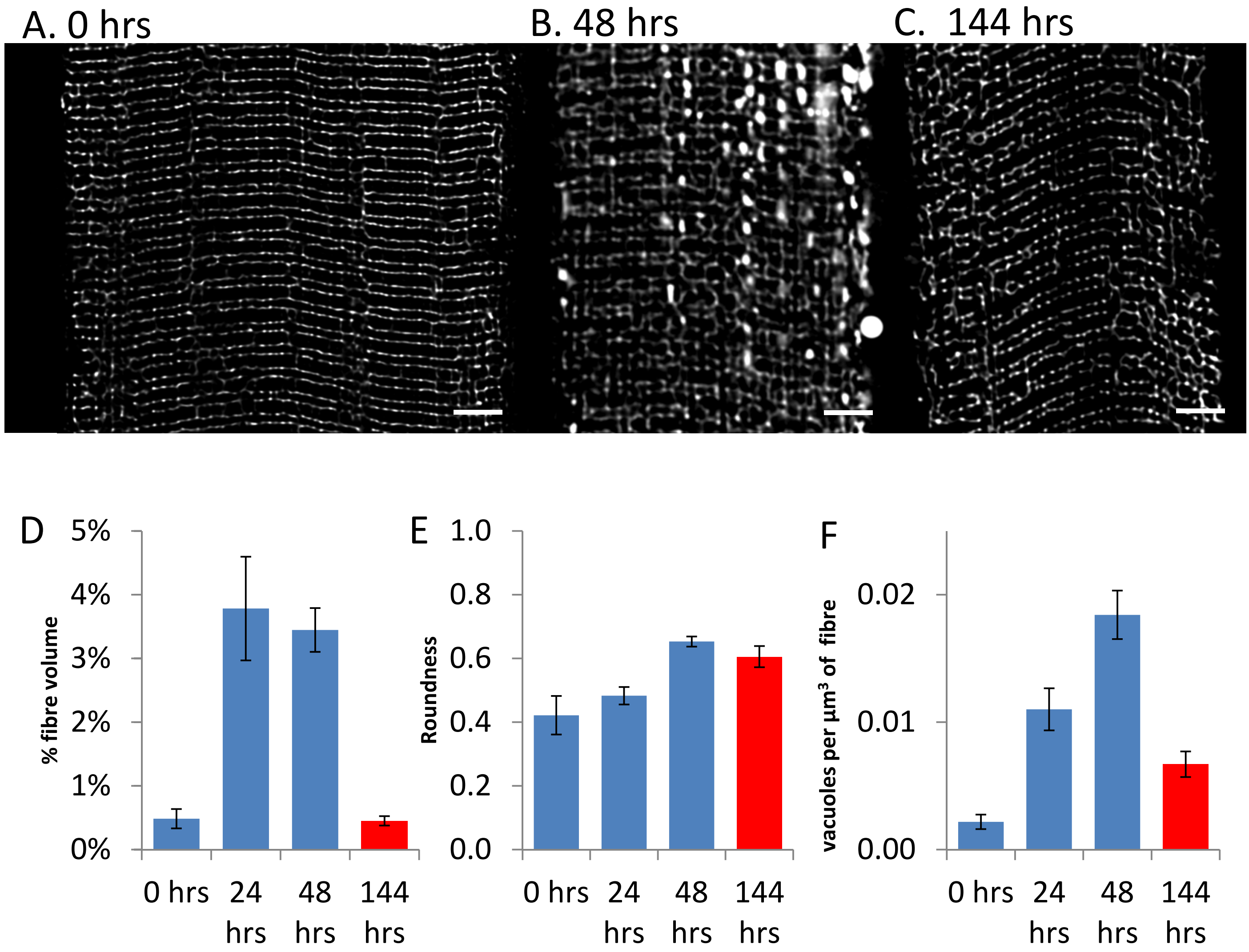

Supplementary Figure 4: Recovery of t-system structure 144 hours following exercise. Comparison of single optical planes from deconvolved confocal z-stacks from muscle biopsies at (A) 0 hours (i.e. prior to exercise), (B) 48 hours and (C) 144 hours illustrates that the overall structure of the t-system is largely restored at the latter time point. While the vacuoles appear fewer in number, the overall transverse regularity of the t-system is not completely similar to the pre-exercise structure. The bar plots illustrate the statistical comparisons of the vacuole measurements at the 144 hrs time point (red bars) which show a near complete restoration of the (D) percentage of the fibre volume that are vacuoles. (E) The vacuoles that remain retain the rounder shape, similar to the 48 hrs time point, (F) however, number of vacuoles per unit volume is >2-fold higher than the pre-exercise fibres. Error bars report SEM; n=33 datasets for the 144hrs time point. Scale bars: 5 µm.

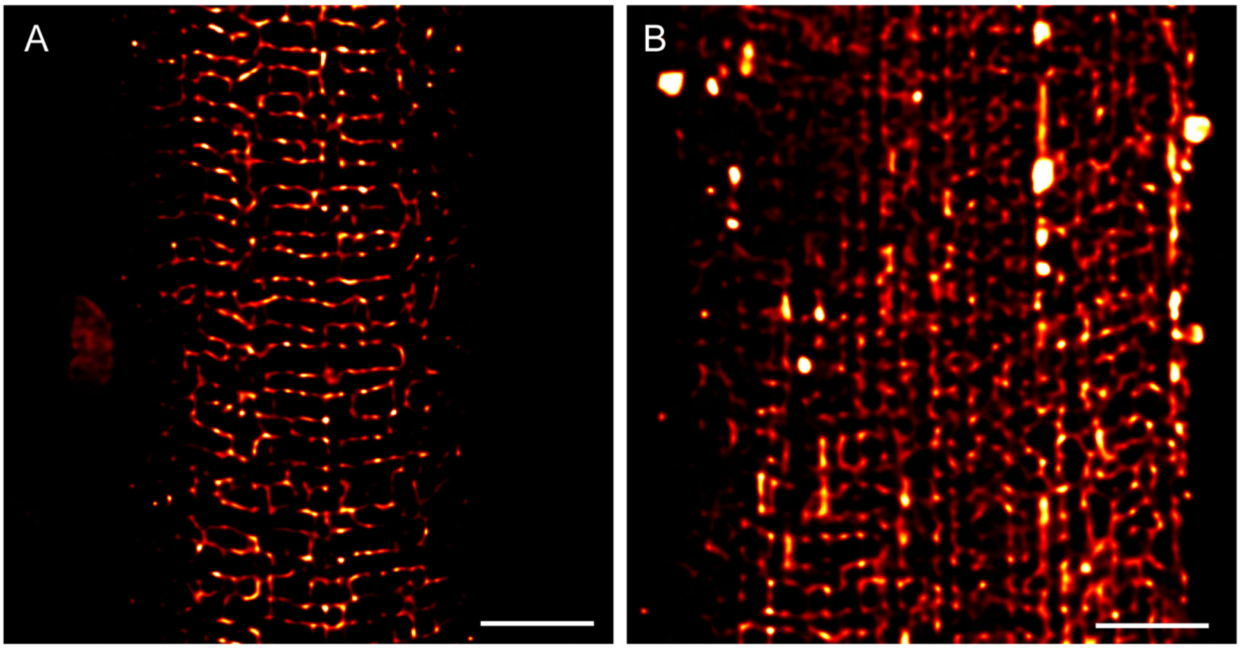

Supplementary Figure 5: Change in the sub-sarcolemmal tubules following exercise. Confocal optical sections from deconvolved z-stacks illustrating the structure of the sub-sarcolemmal tubules in a (A) pre-exercise (0 hrs) biopsy and a (B) biopsy 24 hrs following exercise . Sub-sarcolemal network was imaged in the same skinned-fibre preparations used for imaging the deeper t-system. In fibres whose surface sarcolemma was carefully microdissected, we visualised the subsarcolemmal tubule network immediately above the coverslip. Confocal z-stacks were obtained of this full volume in order to visualise these tubules in a glancing optical section at the surface of the fibre next to the coverslip. Whilst there appears to be a non-uniform distribution of the fluorescent tubular volume marker, the prominent vacuoles (bright structures) and a less regular t-system structure was apparent in the fibres obtained following exercise. Scale bars: 5 µm.

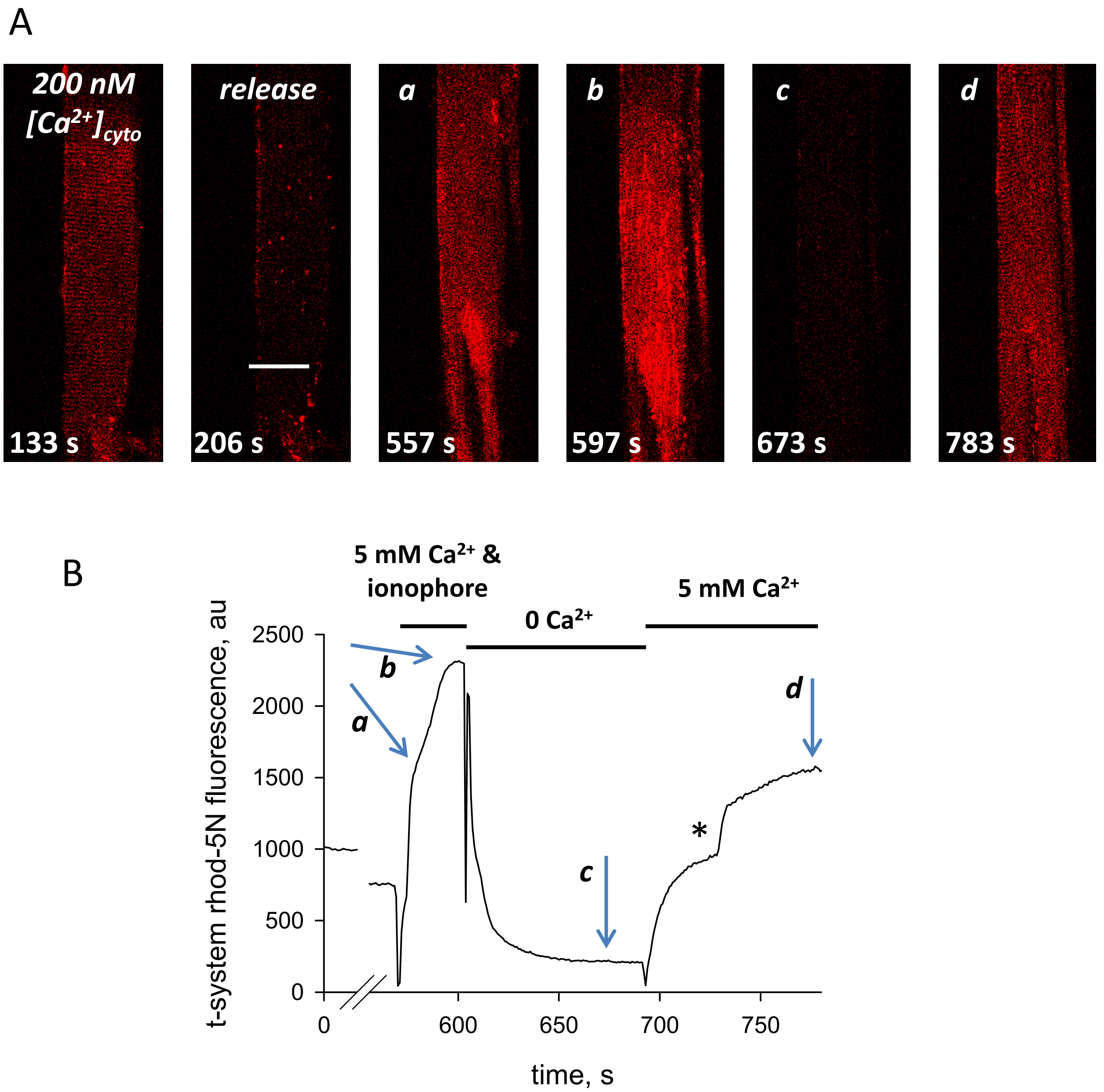

Supplementary Figure 6: Vacuolation and devacuolation occurs within seconds when the t-system is permeable to Ca^{2+}. A, selected t-system rhod-5N fluorescence images of a skinned fibre obtained from a biopsy of a 26 yr old healthy male. First image in standard solution containing 200 nM $[Ca^{2+}]_{cyto}$ shows a regular t-system predominantly made up of transverse tubules. Activation of SOCE in caffeine solution (second image) shows a significant loss of fluorescence signal, indicating Ca^{2+} was lost from the t-system. No vacuolation was observed in the absence of ionophore. The next 4 images, marked *a*-*d*, correspond to the points marked on the spatially averaged profile (B). Upon exposure of the fibre to ionophore and 5 mM Ca2+, the t-system rhod-5N fluorescence signal increases and maintains its transverse structure (*a*). After the point marked *a*, the t-system began to vacuolate, as indicated by ***b*.** This increase the overall fluorescence signal. The removal of Ca^{2+} caused a virtual complete loss of fluorescence signal form the t-system (*c*). The readdition of 5 mM Ca^{2+} to the bathing solution caused the t-system rhod-5 fluorescence signal to increase to display only a transverse pattern, indicating that the vacuoles were lost in the absence of Ca2+. *, indicates an adjustment of the optical imaging slice. Scale bar: 35 µm.

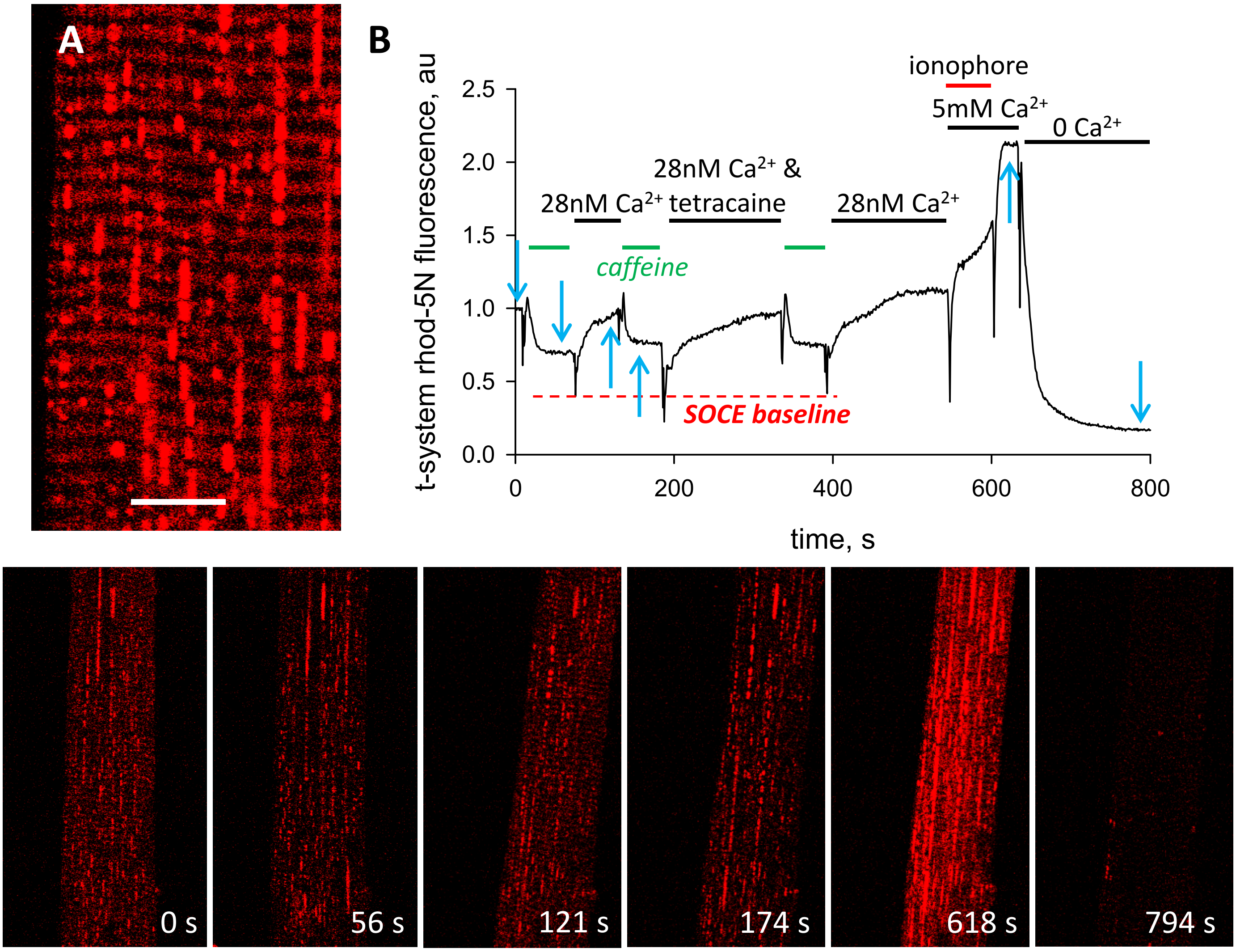

Supplementary Figure 7: Mild vacuolation induces a composite response of an initial spike of $[Ca^{2+}]_{t\text{-}sys}$ (see Fig 5) followed by a partial depletion of $[Ca^{2+}]_{t\text{-}sys}$ (see Fig 3) in the presence of caffeine. A, relatively high resolution image of the t-system rhod-5N fluorescence showing transverse tubules and longitudinally oriented vacuoles containing Ca^{2+}. The image in A was captured prior to the experiment conducted in B. B, spatially-averaged profile of a xyt series during the exchange of internal solutions containing Ca^{2+} to release SR Ca^{2+} (caffeine) and load Ca^{2+} back into the SR and t-system. At the end of the experiment ionophore is used to permeabilize the t-system to Ca^{2+} and the fibre was exposed to 5 mM and 0 Ca^{2+}. Images at the bottom are taken form the xyt series as indicated by the time-stamp and blue arrows on B. The presence of vacuoles is persistent through all images except that in the presence of 0 Ca^{2+} following ionophore exposure. In the presence of caffeine, the transverse tubules became less obvious consistent with the reduction in the average t-system fluorescence signal (see Fig 4). The SOCE baseline in B has been determined as the average t-system fluorescence from an area of the fibre devoid of vacuoles whilst in the presence of caffeine. Note that this baseline is lower than the baseline when more vacuoles were present (Figs 4 & 5). Scale bar: 10 µm.

Supplementary Methods

The datasets used for Figs 1 & 2 of the paper are derived from fibres from training experienced men. The training protocols used is described in Supplementary Table 1 and the description of the men participating in the training session is shown in Supplementary Table 2.

Some participants in these training sessions were exposed to a cold water treatment for recovery from training), as described in detail [2]. Two of three individuals in Study 2 were subjected to cold water treatment post exercise (results in Fig 2). Three of six participants were exposed to cold water treatment post exercise in Study 1 (results in Supplementary Fig 4). No difference was observed in the t-system structure from either group within the two Studies, so the data was pooled. It is expected that cold water immersion would lower the temperature of the muscles, which may affect the rate that the Na^+-K^+ ATPase was able to function and thus may have a minor, if any, effect on the size of vacuoles within the muscles post-eccentric contractions [3].

Supplementary Table 1: Training session composition for acute and chronic exercise.

Training session composition of studies 1; training, and 2; acute.

	Study 1.		
**Exercise**	**Repetitions (in set order)**	**Repetition load**	**Progression**
**1. 45° leg press**	Wk 1-2; (8, 8, 10, 12, 10, 10)<br>Wk 3-4; (8, 8, 10, 12, 10, 10)<br>Wk 5-6; (8, 8, 10, 12, 10, 10)<br>Wk 7-8; (8, 8, 10, 12, 10, 10)<br>Wk 9-10; (8, 8, 10, 12, 10, 10)<br>Wk 11-12; (8, 8, 10, 12, 10, 10)	8-, 10- and 12-RM<br>8-, 10- and 12-RM<br>8-, 10- and 12-RM<br>8-, 10- and 12-RM<br>8-, 10- and 12-RM<br>8-, 10- and 12-RM	Adjusted at the start of every 5th session
**2. Knee extensions**	Wk 1-12; (12, 12, 12)	12-RM	Adjusted at the start of every 5th session
**3. Knee flexions**	Wk 1-12; (12, 12, 12)	12-RM	Adjusted at the start of every 5th session
**4. Walking lunges**	Wk 1, 4, 7, 10; (10, 12, 14)<br>Wk 2, 5, 8, 11; (12, 14, 16)<br>Wk 3, 6, 9, 12; (14, 16, 18)	Week 1–3; 20% of PTBM<br>Week 4–6; Week 1-3 load + 5 kg<br>Week 7–9; Week 1-3 load +10 kg<br>Week 10–12; Week 1-3 load +15 kg	-
**5. Plyometrics**	Wk 1-3; (12, 12, 12)<br>Wk 4-6; (12, 12, 12)<br>Wk 7-9; (12, 12, 12 each leg)	Body weight<br>50% of lunge load<br>50% of lunge load	Countermovement drop jumps<br>Slow eccentric squat jumps<br>Split lunge jumps

	Wk 10-12; (12, 12, 12)	50% of lunge load	Countermovement box jumps
	**Study 2.**		
**1. 45° leg press**	(8, 8, 10, 12, 10, 10)	8-, 10- and 12-RM	-
**2. Single leg squat**	(12, 12, 12)	12-RM	-
**3. Knee extensions**	(8, 8, 10, 12, 10, 10)	8-, 10- and 12-RM	-
**4. Walking lunges**	(12, 12, 12)	12-RM	-

Exercise order is denoted by 1–5 (study 1) and 1–4 (study 2). Concentric phases of repetitions were performed as quickly as possible, whilst eccentric and inter-repetition periods lasted approximately 1 sec. Recovery time between sets was set at 1 min, and recovery between exercises was set at 3 min, leading to total training session duration of approximately 45 min. RM; repetition maximum, PTBM; pre-training body mass.

Supplementary Table 2: Description of participants in exercise training sessions.

	Study 1	Study 2
Age (years)	20.8 ± 2.32	23.7 ± 2.52
Height (m)	1.83 ± 0.08	1.81 ± 0.06
Body mass (kg)	75.3 ± 7.8	83.5 ± 3.52
45º leg press 1-RM (kg)	346 ± 107	306 ± 31
Knee extension 1-RM (kg)	82.9 ± 14	68 ± 16

Study 1 & 2 defined in Table 1.

Timing of biopsies.

Acute biopsies were taken at '0' (~30 min before exercise), and then 24 and 48 hours after exercise. In the longitudinal study, the '0' biopsy was collected 4-5 days before the first exercise training session, whilst the 'post-exercise' biopsy was collected 6-7 days after the last training session.

References

1. Jayasinghe, I.D. & Launikonis, B.S. Three-dimensional reconstruction and analysis of the tubular system of vertebrate skeletal muscle. *J Cell Sci* **126**, 4048-4058 (2013).
2. Roberts, L.A. *et al*. Post-exercise cold water immersion attenuates acute anabolic signalling and long-term adaptations in muscle to strength training. *J Physiol* **593**, 4285-301 (2015).
3. Yeung, E.W., Balnave, C.D., Ballard, H.J., Bourreau, J.-P., & Allen, D.G. Development of T-tubular vacuoles in eccentrically damaged mouse muscle fibres. *J Physiol* **540**, 581-592 (2002).
